# Designing healthcare transformation: a hybrid model between the hospital and home for high-risk pregnant women

**DOI:** 10.3389/fdgth.2026.1797655

**Published:** 2026-09-24

**Authors:** Nirit Putievsky Pilosof, Rakefet Yoeli-Ullman, Shlomi Toussia-Cohen, Esther Galler, Galia Barkai, Eyal Zimlichman, Abraham Tsur

**Affiliations:** 1 Coller School of Management, Tel Aviv University, Tel Aviv, Israel; 2 ARC Innovation at Sheba Medical Center, Tel-Hashomer, Israel; 3 Department of Obstetrics and Gynecology, Sheba Medical Center, Ramat Gan, Israel; 4 Gray Faculty of Medical and Health Sciences, Tel Aviv University, Tel Aviv, Israel; 5 Sheba Beyond Virtual Hospital, Sheba Medical Center, Tel-Hashomer, Israel; 6 Central Management, Sheba Medical Centre, Ramat Gan, Israel; 7 The Dina Recanati School of Medicine, Reichman University, Herzliya, Israel

**Keywords:** digital transformation, healthcare design, high-risk pregnant women, hospital at home, hybrid model, remote care, tele-monitoring, people-process-place (3P) framework

## Abstract

**Introduction:**

Healthcare organizations are developing hybrid models of care between the hospital and home using remote technologies for monitoring and communication, aiming to transform their healthcare service. While most programs focus on technology integration and adaptation, or on clinical safety and quality, sustainable healthcare transformation requires a system design approach that coordinates change across human, technological, and spatial dimensions. This study investigates a novel hybrid model for women with high-risk pregnancy, offering an alternative to prolonged hospital stays. The model enables home hospitalization through intensive telemonitoring and daily remote clinical encounters, complemented by twice-weekly in-person hospital assessment by the same clinical team. Advanced telemedicine technologies enable women to perform essential maternal and fetal assessments at home under remote guidance, whereas inpatient visits support evaluations that are not yet feasible remotely. The People-Process-Place (3P) framework, which conceptualizes healthcare systems as dynamic configurations of social actors, care processes, and physical and digital places, provides a useful lens for examining the hybrid model as a form of healthcare transformation that simultaneously reshapes human roles, organizational workflows, and care environments.

**Methods:**

The qualitative study, based on observations and forty interviews of patients, staff, management, and technology directors, examines the transformation of the hybrid model of care by adopting the People-Process-Place (3P) framework to analyze the multidimensional change.

**Results:**

The findings demonstrate transformation across all dimensions of the People-Process-Place framework. The hybrid model reshaped professional practice, enhanced patient autonomy, and increased family involvement (People), thereby expanding relational continuity of care. It reconfigured the patient journey, introduced new patterns of technology use, redefined organizational policies and advanced economic models (Process), and expanded care across hospital and home settings, with physical and digital environments provided in diverse locations (Place). At the intersection of People-Process-Place lay safety, trust, and experience, which affect the system's flexibility and ability to manage challenges and scale.

**Conclusions:**

The study presents a design framework for healthcare transformation to examine the intersection of human, technological, and spatial change. The case of the hybrid model for high-risk pregnant women demonstrated the complexity of transformation and the need for a design framework to evaluate and develop change processes.

## 1 Introduction

Healthcare systems are undergoing accelerated transformation in response to mounting demographic, clinical, and organisational pressures. Rising demand, workforce shortages, and financial constraints have exposed the limitations of hospital-centric models that concentrate care within institutional boundaries (1–4). Concurrently, advances in digital health technologies, such as telehealth, remote patient monitoring, and artificial intelligence (AI), have expanded the technical capacity to deliver care across settings (5, 6). However, a growing body of evidence indicates that technological innovation alone is insufficient to achieve sustainable healthcare transformation (7, 8). Instead, new models of care require systematic approaches that address the interdependencies between human experience, organisational processes, and environmental design (4, 9).

The need for a systemic approach is also evident in maternity care for high-risk pregnant women. High-risk pregnancies often necessitate frequent monitoring, specialist oversight, and timely escalation, traditionally resulting in prolonged hospitalisation. While hospital-based care offers clinical safety, it can disrupt daily life, increase stress and anxiety, and place substantial strain on inpatient capacity (10, 11). Hybrid models of care that bridge the hospital and the home have emerged as promising alternatives, yet their successful implementation depends on more than the deployment of digital tools (12, 13). This paper examines the design of a hybrid model between the hospital and home for high-risk pregnant women, using the People-Process-Place (3P) model to conceptualize healthcare transformation as a multidimensional design challenge.

### 1.1 Healthcare transformation

Healthcare transformation is increasingly recognized as a complex, system-level endeavor rather than a linear process of innovation adoption (14, 15). Despite substantial investment in digital health, many initiatives struggle with non-adoption, abandonment, or limited scalability due to misalignment with clinical workflows, organizational culture, and patient needs (7, 16). Recent reviews of virtual hospital and telemedicine initiatives reinforce that successful transformation depends on coordinated change across governance, workforce, workflows, and care environments, not merely the availability of digital infrastructure (17–19).

Socio-technical perspectives emphasize that health systems are complex adaptive systems in which changes to technology inevitably reshape roles, responsibilities, and relationships (20, 21). Transformation, therefore, involves reconfiguring how care is organized, experienced, and governed, including professional boundaries, accountability structures, and care pathways. Importantly, it also entails a spatial reorganization of care as services extend beyond hospitals into homes and digitally mediated environments (22). Without a systematic design approach, digital transformation risks increasing clinician burden, fragmenting care, or exacerbating inequities, even when clinical outcomes appear favourable (23).

In maternity care, the consequences of poorly designed transformation are particularly significant. Pregnancy is not only a clinical condition but an embodied, emotional, and social experience. High-risk pregnancy intensifies uncertainty and surveillance, making trust, continuity, and relational care central to safety and wellbeing (24, 25). Designing new care models in this context, therefore, requires frameworks that can integrate clinical effectiveness with experiential, organizational, and spatial considerations.

### 1.2 Hybrid models between the hospital and home

Hybrid models of care, commonly described as hospital-at-home, virtual wards, or digitally enabled care pathways, have gained increasing attention as mechanisms for delivering hospital-level care outside institutional settings. The concept of a “Hospital without Walls” (26, 27) is enabled by telehealth, remote monitoring technologies, and interoperable data platforms, distributing care across settings. Recent evidence demonstrates that hybrid and virtual care models can maintain or improve clinical outcomes while enhancing patient satisfaction and reducing burden on health systems (6, 28–30).

In maternity care, hybrid approaches have been explored for diverse high-risk medical conditions (31–35). Recent pilot and feasibility studies show that remote maternal fetal monitoring, including home-based cardiotocography and physiological monitoring, is clinically feasible, acceptable to women, and capable of producing data comparable to in-clinic assessments. Systematic and scoping reviews further indicate that hybrid prenatal care models can preserve safety while improving convenience, autonomy, and engagement for both low and high-risk populations (36–40).

However, hybrid care also increases the complexity of healthcare delivery, which is provided across multiple locations, can be synchronous or asynchronous, and often involves extended diverse personnel (41). Hybrid care also redistributes work and responsibility (42, 43). For example, researchers found that home-based telemonitoring requires new, proactive roles among midwives, obstetricians, and monitored women, introducing a fine-tuned balance between personalized and standardized care to provide safe, optimal monitoring of high-risk pregnancies (44). Moreover, research shows that the home is not a neutral extension of the hospital. Variations in housing conditions, privacy, social support, and digital connectivity shape both the feasibility and equity of hybrid care (45, 46).

These challenges underscore the need to move beyond quantitative, outcome-focused evaluations toward qualitative design-oriented approaches that account for how hybrid models are enacted across diverse contexts. In high-risk pregnancy, where safety, quality of care, experience, and equity are tightly intertwined, such approaches are essential.

### 1.3 Designing the intersection of human, technology, and space

Healthcare transformation in hybrid care models entails change across human, technological, and spatial dimensions. The 3P model, integrating change in People, Process, and Place, offers a structured framework for understanding and designing healthcare transformation as an integrated socio-technical-spatial system (47–49). It provides an interdisciplinary inquiry into technology-enabled care, bridging human experience, system performance, and the evolving geographies of healthcare delivery. Emerging from healthcare design, organisational change, and patient experience research, the model conceptualises care delivery as the interaction among human actors (People), organisational and clinical workflows (Process), and the physical and digital environments in which care occurs (Place). Rather than treating these dimensions in isolation, the 3P model emphasises their interdependence and co-evolution (50).

The **People** dimension in healthcare foregrounds patients, families, clinicians, and support staff, focusing on lived experience, capability, trust, and wellbeing. In hybrid maternity care, this includes women navigating risk and uncertainty, informal caregivers supporting care at home, and clinicians adapting to remote, data-driven forms of practice. The **Process** dimension encompasses care pathways, protocols, information flows, technology use, organisation management, economic models, and governance mechanisms. Hybrid models require substantial redesign of processes, such as monitoring, escalation, documentation, and coordination across institutional and domestic settings. The **Place** dimension encompasses healthcare facilities and extends beyond the hospital to include homes, community settings, and virtual environments. In hybrid maternity care, the home becomes a site of clinical surveillance, decision-making, and emotional labour, integrated with digital infrastructures that mediate this new geography of care.

By integrating People, Process, and Place, the 3P model provides a robust framework for designing healthcare transformation and evaluating its development, implementation and outcomes. This paper employs the 3P model to examine the design of a hybrid hospital-home model for high-risk pregnant women, thereby contributing to contemporary digital health scholarship on transformation design.

## 2 Methods

The exploratory study employed a qualitative approach to examine how the hybrid care model for high-risk pregnant women demonstrates the complexity of designing for healthcare transformation. We explored the evolving model of care through our ongoing research from October 2024. The qualitative study is based on participant observation and forty formal interviews with patients (14), patients' spouses (2), medical and nursing staff members (13), managers from Sheba MC and Sheba Beyond (6), and remote care companies (5). Participants were purposively identified because of their direct involvement in the hybrid program and were interviewed after they consented to participate in the study. The interviews included medical and nursing staff members involved in the program, as well as hospital managers and directors of the remote care companies. Patients and spouses were recruited based on their availability and interest in participating in the study. The main researcher observed remote sessions from the hospital with patients at home, such as physician checkups, midwife monitoring, and technician-performed ultrasound. Additionally, patients were followed during their visits to the hybrid unit at the hospital. The observations were documented in field notes and, when possible, recorded and transcribed. The researchers also utilized interview notes, field observation photos, architectural drawings of the medical ward and hybrid unit, webinars presenting the model, and media coverage.

The formal semi-structured interviews with patients covered topics such as their experiences of the hybrid hospitalization, receiving care both in hospital and at home, the use of telemedicine technologies, interactions with medical staff, and the impact of the service on their families and daily lives. The interviews were based on the patient's specific condition and home situation, and on their willingness to share information. The semi-structured interviews with staff members, hospital management, and technology directors addressed topics related to changes in professional practice, management of operations, organization policy, and economic barriers. Beyond the specific questions for each participant group, the interview questions also explored the potential of the hybrid model of care, the experiences of all users, including patients, staff, and family, and opportunities for future development. All the interviews were conducted by the main researcher. Most interviews were conducted in person at the hospital, with some held virtually via Zoom. Each interview lasted approximately 30–60 min, primarily recorded in Hebrew with the consent of the interviewees, transcribed, and professionally translated into English.

Following the data collection process, thematic qualitative data analysis was employed to identify emerging themes from the interviews and observations based on principles of naturalistic inquiry and a grounded approach to conceptual development (51–53). The interview transcripts and observation records were read through twice for familiarization before coding. The coding process combined inductive identification of themes with a deductive organization based on the 3P framework, grouping emerging themes within the People, Process, and Place dimensions. Two authors independently reviewed the coded material, discussed differences, and reached consensus on themes related to the 3P model. Similar ideas were clustered through the field notes and interview coding, with supporting quotes provided as evidence for the case analysis (54). The emerging themes were characterized and divided by the 3P model. The data segments within these thematic clusters were then assessed by a third member of the research team to examine consistency between the source material, coding, and interpretation. Triangulation across interviews, observations, field notes, and contextual materials was used to strengthen analytic credibility. At the end of the data analysis phase, the validity of the themes was checked and confirmed with clinical and management leaders with oversight of the care processes.

## 3 The case of Sheba beyond: hybrid care for high-risk pregnant women

This study investigates a novel hybrid model for pregnant women with severe maternal and/or fetal complications of pregnancy. The model provides an alternative to prolonged hospital admission indicated for maternal and/or fetal intense monitoring, which often lasts weeks or even months before delivery. The hybrid model allows eligible high-risk pregnant women hospitalization at home, receiving hospital-level perinatal care at home by integrating intense remote surveillance, including clinical assessment 3 times daily, as well as fetal heart rate and biophysical monitoring, with twice-a-week physical visits of the women to the hospital unit (40).

Eligibility criteria for the hybrid program include a maximal distance of 35 km from Sheba MC or collaborating centres and full compliance with the program requirements. The medical criteria include a GA > 23 + 0, no vaginal bleeding, and no need for vaginal examination. Contraindications that are indication-specific are severity features in women with hypertensive disorders of pregnancy, umbilical artery absence in women with fetal growth restriction, and bile acids > 100 in women with intrahepatic cholestasis of pregnancy.

The intensive remote care at home leverages cutting-edge telemedicine technologies to replace the standard hospital daily assessment, including vital signs, glycemic control (Datos Health), fetal heart tracing (INVU by Nuvo™), and fetal ultrasound (Pulsenmore™), performed by the woman with remote online guidance of the caregiver. Before leaving the hospital, the patients receive the technology devices and are trained to use them remotely. Only after the patient feels confident and proves the ability to manage the technologies can they leave the hospital unit for their home.

When visiting the hospital twice a week for on-site checkups, the women are treated in the hybrid unit within the high-risk medical ward by the same medical and nursing team responsible for remote home care. The physical hybrid unit at the hospital is designed as an outpatient unit with space for five patients and their companions, a nurse station, and a separate bathroom.

Between October 2023 and December 2025, the program recorded 206 admissions, resulting in 2,998 days of intensive remote care, with an average of 14.6 admission days per patient. During this period, there was only one event of noncompliance to return urgently to admission [for fetal nonstress test (NST) indication], and zero adverse events at home.

## 4 Results

The analysis of the data collection revealed that transforming the model of care for high-risk pregnant women into a hybrid model between the hospital and the home required changes at multiple levels. The study exposed the complexity of changes in the behavior of the People involved (staff practice, patient autonomy, and family involvement), changes in the Process of healthcare (the patient journey, technology use, and the organization policy), and changes related to the Place of care (home vs. hospital, digital vs. physical, and the provision of care from diverse locations) (Figure 1 and Table 1).

**Figure 1:**
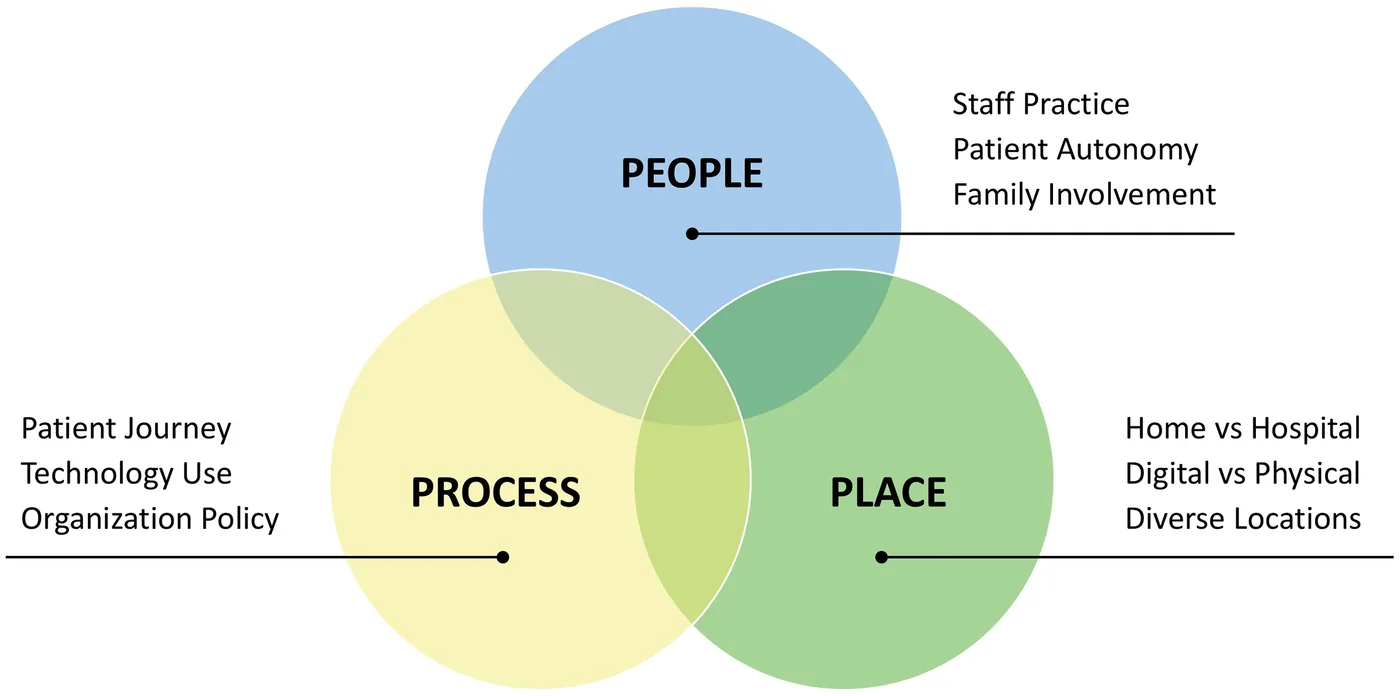
Emerging themes in the design of the hybrid model of care between the hospital and home.

**Table 1:** Data analysis identifying emerging themes related to the people-process-place model.

Overarching themes	First-order themes	Second-order themes
Change in PEOPLE	Staff practice	Redefining professional roles and responsibilities
		Seeking relational continuity of care
		Rearranging collaborative teamwork
	Patient autonomy	Enhancing patient choice
		Relying on patient engagement
		Supporting patient well-being
	Family involvement	Maintaining family life
		Empowering motherhood
		Mitigating community and cultural inequalities
Change in PROCESS	Patient journey	Augmenting hybrid pathways
		Defining schedules and routines
		Enabling continuity of work and study
	Technology use	Training and assistance for patients and staff
		Enhancing the reliability of remote technologies
		Managing data security and privacy
	Organization policy	Redefining protocols and regulations
		Managing hospital operations
		Developing Value-Based Payment (VBP) models
Change in PLACE	Home vs. hospital	Expanding accessibility to care
		Enhancing personalization and comfort
		Mitigating patient community support
	Digital vs. physical	Enhancing the convenience of care
		Fostering personal connections
		Redefining the privacy of patients and staff
	Diverse locations	Facilitating patients active lifestyle
		Managing the lack of staff and preventing burnout
		Enabling family and friends participation

### 4.1 Change in PEOPLE

#### 4.1.1 Staff practice

##### 4.1.1.1 Redefining professional roles and responsibilities

The shift to a hybrid model brings significant changes to professional roles and responsibilities among healthcare staff. The sense of control provided by hospitalization in the department is contrasted with the challenges and responsibilities of remote care. As one staff member noted:

“It is clear to us that at home and also in the hospital, a fetus could die even if we do a monitor three times a day and a bio-physical ultrasound twice a day, which is the maximum. The fetus can die in between examinations, but hospitalization provides a sense of control, a feeling that we are doing something to identify the situation in time.” (staff interview 10)

Remote care requires staff to be more cautious and to consider each decision about hospital re-admission carefully; as a result, patients also perceive the hybrid model as highly professional and responsible, sometimes even more cautious than traditional hospital care. Yet, resistance to change was evident among some staff, particularly regarding new technologies and remote practices:

“For me, as a nurse, it is very difficult to provide remote care. I have to touch the person; I have to feel them. I have to be able to hug them when they cry. The distance is difficult for me. It is a matter of personality.” (staff interview 8)

Technical and logistical challenges, such as internet connectivity and patient compliance, also impact the professional examination process. The results demonstrate that hybrid care requires staff to adapt to new responsibilities, technologies, and communication styles while maintaining high standards of care and patient safety.

##### 4.1.1.2 Seeking relational continuity of care

The hybrid model reshapes the relationship between healthcare staff and patients. Unlike traditional hospital care, where frequent staff turnover can hinder continuity, the remote model enables patients to be cared for by the same team of physicians and midwives across hospital and home encounters. This organizational continuity enables patients and clinicians to develop relationships over time, fostering trust and deeper connections. As one staff member noted,

“It is important to establish a relationship based on trust, not just a situation of ‘I am the doctor, and I will tell you what to do.’ That connection can be accomplished proportionate to the length of the relationship and the individual surroundings [the home] that add colours to the picture.” (staff interview 23)

Patients in the hybrid program report getting to know nurses and staff well, describing them as partners in their care journey. Remote care also encourages more personal and explanatory communication, because patients actively operate monitoring technologies and clinicians therefore need to explain procedures, observations, and decisions more explicitly:

“Although it is virtual, communication with the medical team is more personal when I am at home than at the hospital unit. They explain more because they rely on my collaboration and operation of the remote monitoring.” (patient interview 6)

Overall, relational continuity was produced by the combination of a consistent care team, repeated remote encounters, and greater patient-clinician collaboration. These mechanisms supported trust and a more personal experience of care despite physical distance.

##### 4.1.1.3 Rearranging collaborative teamwork

The implementation of the hybrid model requires a fundamental reorganization of collaborative teamwork among healthcare professionals. Effective collaboration between doctors and midwives is essential, with experienced nurses playing a critical role in patient safety and monitoring. As one staff member emphasized:

“Medically, professionally, nursing-wise, knowledge, reflexes—nurses are the ones who safeguard us. They know the patients, they pay attention to the monitor night and day. If the nurses aren't proficient with good reflexes and a lot of experience, they will miss more things.” (staff interview 19)

Trust between team members was highlighted as a key factor for success in remote care settings. Overall, hybrid home hospitalization demands experienced, adaptable, and trusting teams, with all members trained in remote care and routines adjusted to support both staff and patient needs.

#### 4.1.2 Patient autonomy

##### 4.1.2.1 Enhancing patient choice

The hybrid model empowers patients by enhancing their autonomy, confidence, and trust in the care process. Patients report feeling secure knowing they can reach out to their care team at any time and return to the hospital if needed:

“I was a little worried, but I knew I was in good hands. I knew I could come back whenever I wanted, and I could call whenever I wanted. I could ask questions and get advice.” (patient interview 19)

Patients and their families recognize that the main purpose of hospitalization is observation, and remote monitoring can provide equivalent reassurance. Accordingly, trust in the care team is central to the decision to participate in home hospitalization and the system's responsiveness further reassures patients. In summary, the hybrid model supports patient choice by providing transparent communication, continuous monitoring, and a strong safety net, while maintaining trust between patients and their care teams.

##### 4.1.2.2 Relying on patient engagement

The hybrid model emphasizes patient engagement and responsibility. Staff and patients alike highlight the necessity for active participation and commitment to ensure the program's success. Staff describe the importance of patient adherence to schedules and rules:

“We had a patient who was never on time when we asked her to connect to the monitor, and she wasn't available for the doctor's call. I told her it's all good and that we aren't angry, but if she can't abide by our few simple rules, it's no problem for her to come back and be re-hospitalized.” (staff interview 23)

Remote care increases patient involvement in the monitoring process, and accordingly, patients report a greater sense of control and understanding. Patients and family members also describe the transition from passive to active roles:

“In the beginning, I found the program frustrating. All of a sudden, I had to be active. In the hospital, I was passive. Staff came and checked me. At home, I was on a schedule like a soldier on duty, with a list of things I had to do.” (patient interview 4)

In summary, the hybrid model shifts responsibility to the patient, requiring high engagement, technical readiness, and active participation to ensure safety and program effectiveness.

##### 4.1.2.3 Supporting patient well-being

The hybrid model provides significant emotional and psychological benefits, enhancing patients' sense of control, reassurance, and connection. Seeing the fetus move during ultrasound at home creates a deeper sense of connection and calm:

“Having a reassurance you can work with, such as an ultrasound scan in which the mother can see the heartbeat and see that the fetus moves, creates a sense of connection and a much calmer feeling, much more so than just having a doctor tell the patient that everything is normal.” (staff interview 23)

Patients describe the comfort and autonomy of home as central to their well-being:

“At home, in addition to the comfort of my own bed, food, and family life, I have a sense of control over my body and pregnancy. Managing the remote monitoring and US helps me connect with my future baby.” (patient interview 4)

Patients also report relief from the isolation and inactivity of hospital stays, finding home life more engaging and restorative. In summary, hybrid home hospitalization supports patient well-being by fostering emotional reassurance, autonomy, and a sense of partnership in care, while enabling patients to maintain comfort and activity in their home environment.

#### 4.1.3 Family involvement

##### 4.1.3.1 Maintaining family life

The hybrid model significantly supports family life by allowing patients to remain at home, stay connected with loved ones, and participate in daily routines. Patients and their families describe the emotional and practical benefits of being together during the challenging period. Remote care at home also makes it easier for partners who often provide emotional support to balance work and family responsibilities:

“It was also easier for my husband to go to work when I was home as opposed to leaving me in the hospital. -Husband: In the hospital, there is a feeling of being alone. At home, she may be physically alone, but there is no fear of leaving her because she is at home.” (patient interview 4)

There is often also a desire to be present for important family moments and holidays. Patients and families also note the practical benefits of maintaining family life:

“My mother and my aunts are very supportive of the home hospitalization. It's not that I can really do anything at home, but it makes it easier on the family unit. It eases the whole logistics of the family unit.” (patient interview 16)

In summary, the hybrid model enables patients to maintain family bonds, participate in daily life, and find emotional support at home, all of which are crucial for well-being during high-risk pregnancies.

##### 4.1.3.2 Empowering motherhood

The hybrid model empowers mothers by enabling them to maintain their maternal roles, stay emotionally connected to their children, and actively participate in family life. It can support the psychological and practical needs of mothers, especially those with young children at home.

“The model supports my mental health. At home, I can take care of my four-year-old son, who couldn't understand why I abandoned him when I had to stay at the hospital. Now he is helping me take care of his fetal sister.” (patient interview 17)

The ability to choose home care is seen as a way to protect children from emotional distress and behavioural problems caused by prolonged separation*.* Mothers who have experienced repeated hospitalizations highlight the emotional relief and stability that home care provides for the entire family:

“My daughter was very affected by my one-month hospitalization during my previous pregnancy. She is older now, and understands more, but still, it was hard for her that I was hospitalized again. Home hospitalization really saved us emotionally: me, my children and my husband.” (patient 20)

In summary, the hybrid model empowers motherhood by allowing mothers to remain active caregivers, maintain emotional bonds, and fulfill their family roles, all while receiving necessary medical care. This approach supports the well-being of both mothers and their children during high-risk pregnancies.

##### 4.1.3.3 Mitigating community and cultural inequalities

The hybrid model helps mitigate community and cultural inequalities by adapting care routines and technologies to diverse religious and cultural needs. For Jewish religious patients, for example, using digital devices on Shabbat and holidays can feel uncomfortable or forbidden at first:

“It will feel strange for a religious person to think of using a telephone on Shabbat and on holidays and using digital technologies at home for the first time, near the children. It may be halachically permissible, but it will take them time to get used to the idea, so meanwhile, they are in conflict.” (management interview 33)

Protocols have been developed to minimize religious and cultural conflicts, especially around Shabbat observance. For many religious women, the challenge is more cultural than religious, considering also community norms around privacy and gender interactions:

“It isn’t that they can’t participate for religious reasons .. It is more of a cultural issue. A woman may feel uncomfortable having to explain to her family and neighbours that the examination she does on Shabbat is permitted, so part of the reason is cultural, and part is psychological and social.” (patient interview 19)

The hybrid model actively addresses community and cultural inequalities by adapting protocols, technologies, and care routines to meet the needs of diverse populations while respecting religious and cultural norms.

### 4.2 Change in PROCESS

#### 4.2.1 Patient journey

##### 4.2.1.1 Augmenting hybrid pathways

In the hybrid model, the patient journey is transformed by integrating both physical (in-hospital) and remote (at-home) modes of care. Patients do not enter home hospitalization directly; instead, they begin as inpatients, allowing staff to assess their clinical and psycho-social needs and ensure suitability for remote care.

“Patients are not admitted directly to home hospitalization. First, they have to be hospitalized as inpatients so we can get to know them and check to see if there are any issues we do not know about… it enables us to get an overall impression of her situation.” (staff interview 1)

Before discharge, patients meet with staff to coordinate expectations and learn about the home procedure. This ensures that patients understand the responsibilities and changes involved in transitioning to remote care. Transitioning home requires successful completion of monitoring and ultrasound tests, demonstrating readiness for remote care. Peer support is built into the process, as new patients meet others who have already experienced the hybrid program, helping to build confidence and understanding. Staff emphasize that technical failures or health deterioration may require patients to return to the hospital, highlighting the importance of flexibility and safety in the hybrid pathway. The remote technologies that are used for home care can be utilized also for inpatient care in case a patient is readmitted. It showed potential to save time and reduce logistical burdens for both patients and staff.

“I can do the US while she is in her patient room at the hospital and I am in the hybrid unit… She was re-hospitalized, and she has all the equipment. First, it saves her from having to go to the ultrasound room and wait outside with all of the other patients. It also helps the technician… She has one less physical examination to do. It gives her more time.” (staff interview 5)

In summary, the hybrid model augments the patient journey by blending physical and remote care, requiring careful assessment, training, and ongoing support. It necessitates changing the process of delivering care.

##### 4.2.1.2 Defining schedules and routines

The hybrid model requires careful coordination of schedules and routines to balance medical needs with patients' personal and family obligations. Staff emphasize the importance of setting expectations early, explaining that home hospitalization requires a strict routine and a daily schedule similar to inpatient care. Staff coordinate remote monitoring times according to hospital routines and staff availability. They try to provide flexibility when possible.

“The morning monitor has to be done at about 7:30, when many women take their children to school.. We did not choose 7:30 as an arbitrary hour. It is an hour of the day that gives us [medical team at the hospital] leeway to manage issues if there is a problem.” (staff interview 1)

The twice-a-week hospital checkup is also challenging for some women, who must arrive early in the morning to meet the program requirements.

At home, patients appreciate the convenience of knowing their exact appointment times for remote tests, which contrasts with the uncertainty and long waiting times typical at the hospital. However, some find the required monitoring periods, in the mornings and afternoons, challenging to manage alongside family responsibilities.

“Sometimes the timing is difficult, having to do the monitor at 16:30 … When your daughter comes home from school? Yes. It's okay, I manage, but I often have to be on the monitor for two hours, and it's a bit challenging” (patient interview 9)

In summary, defining schedules and routines in the hybrid model involves balancing medical protocols with patients' personal lives, providing flexibility where possible, and maintaining clear communication to ensure safety and adherence.

##### 4.2.1.3 Enabling continuity of work and study

The hybrid model allows patients greater flexibility to continue work and study from home, in contrast to the limitations experienced during hospital stays.

“It was very hard for me to be hospitalized at the hospital… I am a high-energy career woman. I tried to work, having Zoom meetings on my laptop, sitting on the bed, but you are in a room with another person… It drove me crazy to sit there and do nothing.” (patient interview 22)

In contrast, patients in the hybrid model report that visits to the hybrid unit and being at home provide more opportunities for personal time, relaxation, and even work. To support continuity of work, staff try to schedule monitoring times to minimize interference with work and family obligations:

“One of our considerations was that a woman hospitalized at home may want to go to work. If we scheduled the monitor for 10:30, instead of 7:30, it would interfere with her workday.” (staff interview 1)

However, there is still a misconception among colleagues that home hospitalization is less demanding, when in reality, it requires significant time and commitment. In summary, the hybrid model offers patients more autonomy and the possibility to continue work or study from home, compared to the restrictions and lack of privacy experienced during hospital stays.

#### 4.2.2 Technology use

##### 4.2.2.1 Training and assistance for patients and staff

The transition to a hybrid model relies on the ability of both patients and staff to adapt to new technologies. Success depends on effective training and engagement with digital tools. As one manager explained:

“The program succeeds or fails according to patients’ ability to control the technologies… adaptation and training are very significant.” (management interview 11)

Staff engagement and leadership play a crucial role in overcoming technological challenges and supporting program implementation:

“The engagement of technologists and program leaders… increases the program's potential. Sometimes people complain the technology is complicated, but having motivated leaders makes a difference.” (management interview 18)

Patients in the program are generally motivated and capable of learning new technologies, which supports the program's success. Patients receive devices and instructions, sometimes with real-time guidance from clinicians, which improves the quality of remote examinations.

“High-risk pregnancy includes the most premium population.. young women who are willing to make an effort, even if it is more complex, for their pregnancy. The fact is, that it succeeds.” (management interview 18)

In summary, the hybrid model's effectiveness relies on comprehensive training, patient motivation, and ongoing support from engaged staff, ensuring that technology enhances care without compromising safety or quality.

##### 4.2.2.2 Enhancing the reliability of remote technologies

The reliability of remote technologies in the hybrid care model is built on trust, validation, and clear division of responsibilities. Staff and management emphasize that trust in the method and technology is essential, especially since pregnancy care is highly litigated and safety concerns are real.

“People have to trust the method and the technology because this is a new system.. First of all, that it is safe. That it is effective. It is useful, comfortable and enjoyable.” (management interview 12)

However, introducing new technology is a complex process that requires careful selection and significant resources. Once a product is stabilized, the team avoids frequent changes. Medical responsibility and legal concerns remain significant barriers to adopting innovative technologies. Overall, the successful integration of remote technologies depends not only on technical solutions, but also on building trust, ensuring safety, and addressing legal and professional concerns among staff and patients.

##### 4.2.2.3 Managing data security and privacy

The transition to a hybrid care model introduces new challenges and requirements for data security and privacy. The program's digital infrastructure is designed to support advanced diagnostics and AI-based decision support, emphasizing the importance of secure data management. Strict protocols and regulatory oversight are required before introducing new technologies, including approval from dedicated committees.

“When we introduce digital technology to the hospital, it has to go through a committee that determines if the program is secure enough. .. Without that, we are forbidden to pass on personal data.” (management interviews 12)

Currently, the lack of integration between digital platforms complicates clinical documentation and access to patient data. Ensuring ease of use for clinicians and patients and maintaining robust cybersecurity are ongoing challenges.

“Currently, the doctors have to move back and forth between four different systems. They have to open different dashboards and documents. In my opinion, that is not scalable, not for the hospital or any other organization.” (technology expert interview 26)

In summary, managing data security and privacy in the hybrid model requires rigorous regulatory oversight, integration of digital platforms, and ongoing adaptation to technical and workflow challenges, all while maintaining clinician efficiency and patient safety.

#### 4.2.3 Organization policy

##### 4.2.3.1 Redefining protocols and regulations

The hybrid model required the development of new clinical protocols and regulations to ensure patient safety, operational efficiency, and alignment with hospital and national policies. Staff emphasize the importance of clear procedures for patient eligibility, emergency situations, and coordination with collaborating hospitals.

“Gynecology is a field that is very medically litigated.. It's frightening. The whole topic of risk management—we have a doctor whose entire role is risk management.” (staff interview 28)

Accordingly, eligibility for the program is limited by distance and the ability of collaborating hospitals to provide immediate care if needed.

“A woman can only be part of the program if she is within a 35 km distance from the hospital or from one of our collaborating hospitals. If we want to expand… we must be certain that the other hospital works according to the same process as Sheba.” (staff interview 1)

Protocols also define how to transfer information and manage readmission:

“The protocol defines how to transfer information if a patient returns. How to inform the staff. A woman discharged to home hospitalization who has to be re-admitted is supposed to go directly to the department.” (management interview 12)

The model's implementation depended on gradual, research-supported change and strong leadership. In summary, redefining protocols and regulations was essential for the safe and effective operation of the hybrid model, requiring careful planning, clear eligibility criteria, and ongoing adaptation to clinical, organizational, and regulatory needs.

##### 4.2.3.2 Managing hospital operations

The hybrid model required significant changes in hospital operations, affecting patient flow, staff responsibilities, and departmental coordination. The hybrid approach enables the department to maintain full occupancy with the most serious cases, while other patients receive care through the hybrid model. This supports both patient priorities and system efficiency.

“If we are able to keep our department at 100% occupancy all the time, caring for the most serious cases, while the rest receive care through home hospitalization, it is a win-win situation for the woman.. But it is also a win for the system.” (Staff interview 10)

Once patients transition to home hospitalization, the department loses daily oversight, regaining responsibility only upon readmission. This creates challenges in tracking patient status and coordinating returns. Beds are no longer reserved for hybrid patients; readmission depends on bed availability, otherwise patients must wait in the ER.

“We have 39 beds, and we are full. According to protocol, if a woman has to be re-admitted, she can come directly to us and not go through the ER only if we have an empty bed. If we don’t have a bed, she goes to the ER and waits there.” (Staff interview 10)

In summary, managing hospital operations in the hybrid model involves optimizing department occupancy, ensuring readiness for emergencies, coordinating responsibilities across units, and adapting bed management and patient return protocols to maintain safety and efficiency.

##### 4.2.3.3 Developing value-based payment (VBP) models

The hybrid model was made possible by mitigating economic barriers to reimbursement and developing VBP models. The program has reduced the average number of hospitalization days, which is attractive to the insurance companies. However, they remain cautious about potential funding abuse and the risk of increasing hospital bed numbers.

“We were able to reduce the number of days of hospitalization from an average of 12 to 10 days, so apparently something is being done right.. The HMOs are wary that it could be used to increase hospital beds.” (management interview 11, 12)

A major challenge is that the insurance companies hold the authority to approve payment for high-risk pregnancy care, making it difficult to discharge patients to home hospitalization. Most programs operate as pilots until the insurance companies are convinced of safety and cost-effectiveness. The hybrid model's requirement for weekly hospital visits serves as an anchor for both clinical and economic reasons, helping prevent abuse of the system and providing control for department directors and the government:

“Hybrid means that they come here twice a week, and that enables us to say that the patients must still come to the hospital. Without that, it is not possible. I think part of the reasoning for hybridity stems from economic constraints as a strategic approach.” (staff interview 28)

In summary, mitigating economic reimbursement challenges in the hybrid model involves navigating funding gaps, overcoming insurance and regulatory barriers, and balancing strategic constraints to ensure sustainability and alignment with the broader healthcare system.

### 4.3 Change in PLACE

#### 4.3.1 Home vs. hospital

##### 4.3.1.1 Expanding accessibility to care

The hybrid model expands accessibility by combining the strengths of hospital-based and home-based care. Patients benefit from scheduled, twice-weekly physical visits to the hospital, which provide direct access to medical staff, advanced monitoring equipment, and a comprehensive basket of services. As one staff member explains:

“In the department, the patient has the monitor available, she can speak to a midwife or just be able to talk to someone on the team about the fetal movements she feels or doesn't feel. The patient is surrounded by the entirety of services we are able to give them in the basket of services we provide.” (Staff interview 10)

Between hospital visits, patients experience the comfort and autonomy of home, supported by remote technologies that enable ongoing monitoring and communication with their care team. This hybrid approach balances the immediacy and breadth of hospital resources with the flexibility, comfort, and personalization of home care. The result is an expanded model of accessibility that optimizes patient experience.

##### 4.3.1.2 Enhancing personalization and comfort

The hybrid model enhances personalization and comfort. Home hospitalization allows patients to remain in their own space, which contributes to a sense of well-being and autonomy.

“Home hospitalization is a game changer. You sleep in your own bed, use your own bathroom and shower, and feel that the staff is with you.” (Patient interview 13)

Patients describe feeling more comfortable and freer at home, able to do things their own way. In contrast, hospital stays can be uncomfortable due to shared rooms, noise, and lack of privacy. Even when the hospital environment is described as pleasant, patients note that it is still not home, and the lack of privacy and comfort can be distressing:

“It was hard for me to sleep the whole time I was at the hospital. First, another patient is in the room. That means there is always some noise, whether it be the woman or the husband.. There was also a lot of turnovers.” (Patient interview 7)

Food and daily routines are also more comfortable at home, with patients able to eat home-cooked meals and avoid the logistics of hospital life. Overall, comfort and personalization are key benefits of home hospitalization, allowing patients to maintain their routines and sense of control.

##### 4.3.1.3 Mitigating patient community support

Patient community support is a nuanced aspect of hospitalization, with both positive and negative experiences reported. Hospitals provide opportunities for social interaction and support among patients:

“There is a certain paradox about being here in the department. On the one hand, you are with people, but on the other hand, it is lonely. I met a lot of women here from all over the country and from all sectors of society and it was very interesting. I heard a lot of stories, some good, some not so good. There are many very nice women here.” (Patient interview 22)

However, hospital stays can also be isolating, as family and friends may not be able to visit regularly, and patients may feel alone despite being surrounded by others. Overall, home hospitalization allows patients to maintain closer contact with family and avoid the emotional toll of hospital isolation. However, it may lack the peer support and shared experience found in the hospital department, which is mitigated by the hybrid unit.

#### 4.3.2 Digital vs. physical

##### 4.3.2.1 Enhancing the convenience of care

The hybrid model blends digital and physical modalities, offering patients flexibility, comfort, and improved access to healthcare services. Digital care, particularly home-based monitoring and ultrasound, is frequently described by patients as more convenient and emotionally rewarding than traditional hospital-based care. One patient explained:

“At home, I see everything. In the department, I can see less. The screen is turned in their direction, so I don't see anything. .. Doing the ultrasound at home is very emotional for me, because I understand what I am doing and understand what I am seeing, so I enjoy it more.” (patient interview 15)

The in-person visits remain important for patient reassurance and clinical oversight. Staff also note that the ability to end remote interactions helps manage logistical overload.

“It becomes a problem, on overload, when the visit doesn't end. It is different when you physically enter the room. When you completed the remote interaction, when you feel that you've exhausted the visit, it's easier to leave.” (staff interview 28)

The hybrid model improves convenience by allowing patients to balance medical care with daily life, combining the flexibility of remote monitoring with the reassurance of regular in-person visits.

##### 4.3.2.2 Fostering personal connections

The integration of digital and physical care in the hybrid model also transforms the nature of personal connections between patients, families, and healthcare providers. Remote care enables more frequent, personalized interactions, often involving family members and providing insights into patients' home environments.

“When you do a monitor in the hospital, the nurse is with you and a lot of other people at the same time. I once waited an hour for the nurse to come. I kept asking my husband to call her, and she didn’t come. You have to wait to be discharged. Here, at home, the nurse is with me all the time. It's a difference between night and day. I feel that the nurse is with me all the time.” (patient interview 13)

While remote care increases accessibility and comfort, staff acknowledge that physical meetings remain important for establishing empathy and understanding subtle patient cues. Overall, the integration of digital and physical care not only improves access and flexibility but also deepens personal connections, supports family involvement, and maintains essential clinical oversight. Patients and staff alike value the balance between remote convenience and the reassurance of in-person contact.

##### 4.3.2.3 Redefining the privacy of patients and staff

The transition to the hybrid model brings new dimensions to patient privacy and the personal experience of care. Interviews reveal that while some patients are sensitive about discussing private issues remotely, others find the experience from home more human and positive.

“There are situations, for example, if discussing very private issues, that I would prefer they ask me if it is okay to talk now or if I would prefer they call back later.” (patient interview 4)

Other patients described feeling comfortable with staff seeing their home, highlighting the personal and humanizing aspects of remote care. While privacy concerns exist, many patients appreciate the increased comfort, personalization, and human connection that remote care at home provides.

#### 4.3.3 Diverse locations

##### 4.3.3.1 Facilitating patients’ active lifestyle

The hybrid model empowers patients to maintain an active lifestyle, integrating medical needs with personal and professional commitments from diverse locations. Patients are able to continue their professional development, such as training as a teacher or studying for a PhD by performing the monitoring and ultrasound scans from their work environments. This flexibility is further reflected in the perception of the program as boutique care, tailored to individual needs:

“When the program is separate from the department, available in different locations, the patients feel that they are receiving a kind of boutique care.” (management interview 12)

Communication with healthcare providers is highly flexible; patients can engage from various locations, not limited to their homes. Connecting from diverse locations often enriches patient-provider relationships, as clinicians gain insights into patients' environments, fostering deeper connections.

“You see their surroundings. One woman speaks to me from the garden with her dog walking around. With someone else, it was obvious from the house and the worn-down sofa that she was not the most well-to-do. Seeing the surroundings is enriching.” (staff interview 23)

The flexibility of remote monitoring from diverse locations empowers patients to maintain autonomy, mobility, and engagement in daily life, but requires adaptation in professional practices.

##### 4.3.3.2 Managing the lack of staff and preventing burnout

Working from diverse locations introduces new opportunities and challenges in managing workforce diversity and preventing burnout. For example, the integration of remote US provides technicians with greater variety in their work environment. As one staff member explained,

“The remote ultrasound for woman at home provides the technicians with diversity at work. It is difficult to work a shift of eight hours in the dark ultrasound room, and the remote care provides flexibility to work also from the office. Managing the technicians’ workforce, I realised this could prevent burnout, so I insisted that all the technicians would be trained and provide both options, physical and virtual US care” (staff interview 3).

International mobility is also possible for staff, as illustrated by a physician who continued working remotely after relocating, acknowledging that the time zone differences benefit program operations.

“I am continuing to work with Beyond remotely from NY, and everything is working well. I begin speaking with patients at 6 AM here, when it is afternoon in Israel, so the time difference serves the program well.” (staff interview 34)

However, the flexibility of remote work requires careful planning and adaptation to maintain a professional atmosphere of care. Remote work also presents technical and logistical challenges and can affect perceptions of professional presence and connection with patients and team members. Overall, the hybrid model offers staff flexibility, diversity, and opportunities for remote work, while also requiring adaptation to new routines, technical challenges, and evolving patient relationships.

##### 4.3.3.3 Enabling family and friends' participation

The hybrid model allows family members to be actively involved in the care routine, regardless of location. Home settings make it possible for the family to participate and support the patient:

“They are basically inviting us into their home. We see different areas of the home, depending on where they are when they speak to us. We see the husband, sometimes the children. This enables the patient's partner to be much more involved. In the hospital setting, they never know when the doctor's visit will take place. At home, they can ask much more significant questions.” (staff interview 1)

The family's ability to connect remotely from diverse locations enhances their sense of partnership throughout the pregnancy. Remote care also supports the family's ability to continue daily work and life commitments.

“I did the ultrasound in different places. My husband and I once had an important appointment in a Ministry office, so when I had to do the monitoring, we found a quiet corner, and he covered me for privacy. I was scared they would send me back to the hospital if I did not do the ultrasound on time.” (patient interview 22)

Overall, the hybrid model strengthens family involvement, enabling loved ones to support the patient and participate in the care process regardless of location.

## 5 Discussion

This study explored the inherent complexity of healthcare transformation through the implementation of a hybrid care model for high-risk pregnant women. By applying the People-Process-Place (3P) framework, the research provides a multidimensional lens to analyze and understand the profound changes required across human, organizational, and spatial domains.

The study results demonstrate how the hybrid model redistributed roles and responsibilities among staff, patients, and family members (People). This is consistent with previous research showing that home-based telemonitoring creates more proactive roles for clinicians and pregnant women (44). While healthcare professionals had to adapt to new practices and overcome resistance to remote technologies, patients gained greater autonomy and responsibility for their care. The hybrid model empowered them to become active participants in their monitoring, fostering a sense of control and well-being, but also requiring high levels of commitment and engagement. Our findings extend previous evidence by showing that this redistribution of responsibility was closely linked to relational continuity, family involvement, and trust in the clinical team.

The hybrid model also transformed the patient journey and healthcare delivery services (Process). The findings demonstrate that hybrid care requires redesign of the care pathway rather than simply transferring monitoring technologies to the home. This supports previous evidence that digital transformation depends on alignment among clinical workflows, organizational structures, governance, and technology (7, 17, 18). In this model, assessment and training before transition to the home, scheduled remote monitoring, regular hospital visits, and escalation protocols formed a coordinated pathway. Successful implementation depended on training, reliable remote technologies, data security, privacy protocols, and organizational support. The model also required gradual, research-supported change, strong leadership, and adaptation to economic and regulatory challenges.

The hybrid model also redefined the location and environment of care (Place). Consistent with previous research, the findings show that the home is not simply a substitute location for hospital care; privacy, family support, digital connectivity, and everyday routines influence the experience and feasibility of hybrid care (45, 46). Combining home-based monitoring with regular hospital visits enabled the model to draw on the advantages of both settings while maintaining access to hospital resources when required. The hybrid model blended digital and physical care, providing flexibility, convenience, and more personal connections through remote technologies, while maintaining essential clinical oversight with in-person visits. Patients and staff could engage in care activities from various locations, supporting active lifestyles and preventing staff burnout. Family and friends could also participate more easily in the care routine.

Beyond illustrating changes across the 3P dimensions, the study highlights their interconnections. The model proved instrumental in dissecting the transformation, revealing how changes in professional roles, patient autonomy, and family involvement (People) are deeply intertwined with redesigned care pathways, technology adoption, and organizational policies (Process), as well as the evolving environments of care, from hospital to home, integrating physical and digital spaces (Place). The interdependencies between People, Process, and Place are evident throughout the study. For example, staff collaboration became essential as teams adapted to new workflows and remote practices from diverse locations. Patient experience was shaped by the intersection of autonomy, technology, and the environment, in which the comfort of home and continuous digital monitoring fostered reassurance and engagement. Flexible protocols and remote access enabled family involvement, helping patients maintain daily routines and emotional bonds.

At the centre of the 3P intersection lie safety, trust, and experience. The hybrid model's success depended on building trust between patients and providers, ensuring safety through rigorous protocols and reliable technology, and delivering a positive experience for all stakeholders. Furthermore, the model demonstrates potential for enhancing flexibility and resource efficiency. By enabling home hospitalisation for eligible patients, the system can optimise hospital bed occupancy, support continuity of care, and help prevent staff burnout, thereby contributing to overall sustainability. The model reveals opportunities for scalable implementation across the healthcare system, offering a blueprint for broader systemic transformation.

## 6 Conclusions

This study demonstrates that successful transformation requires more than the integration of new technologies; it demands a systemic design approach that coordinates change across human, technological, and spatial dimensions. By applying the People-Process-Place (3P) framework to the development of a novel hybrid model for high-risk pregnant women, we have shown how multidimensional change can empower patients and families, transform care delivery, and expand the environments in which care is provided.

The hybrid model described here bridges hospital and home, leveraging telemedicine and intense remote monitoring to offer an alternative to prolonged inpatient stays. This approach not only enhances patient autonomy and well-being but also supports family and community involvement while maintaining intensive clinical oversight. However, the hybrid model demands the transformation of professional roles, new models of collaborative teamwork, patient engagement and shared responsibility with the care team. Managing the hybrid model necessitates redesign of care pathways, technology adoption, and organizational policies, highlighting the challenges and the need for coordinated, sustainable transformation.

The 3P framework provides a structured way to identify and communicate the interdependent changes required for healthcare transformation. Furthermore, the interdependencies among the 3 dimensions—People, Process, and Place—are evident throughout the study, revealing that changes in one dimension depend on and reinforce changes in the others. Safety, trust, and experience lie at the intersection of these dimensions, forming the foundation for successful healthcare transformation. These findings support a systemic, design-driven approach to hybrid care and suggest that future research should examine its implementation and adaptability across diverse clinical, cultural, and economic contexts.

## 7 Limitations

Some aspects of the hybrid program are limited to prolonged admission required for high-risk pregnancy and may not apply to other shorter admission scenarios. The study did not independently evaluate clinical effectiveness or birth outcomes. Member checking was conducted with clinical and management leaders but not with patients or family participants. In addition, exact numbers of eligible and invited participants were not prospectively recorded, preventing calculation of an overall recruitment response rate. More studies on the design of hybrid care services and the implementation of telemedicine technologies in different programs are required, as well as various medical specialities and levels of care. Further research using the People-Process-Place framework in diverse clinical, environmental, cultural, and economic contexts will enhance the knowledge base needed for designing hybrid models of care and healthcare transformation. Future research could also prospectively evaluate the model using an implementation research framework with predefined outcomes, complementing the current design-oriented 3P analysis.

## Data Availability

The datasets presented in this article are not readily available because the IRB approval limits the sharing of the datasets. Requests to access the datasets should be directed to abraham.tsur01@post.runi.ac.il.
